# Geopolymer CLSM with Off-Specification Fly Ash and Bottom Ash: A Sustainable Approach to Hazardous Waste Utilization

**DOI:** 10.3390/ma18133105

**Published:** 2025-07-01

**Authors:** Alexis K. VanDomelen, Ahmed A. Gheni, Eslam Gomaa, Mohamed A. ElGawady

**Affiliations:** 1 Department of Civil, Architectural & Environmental Engineering, Missouri University of Science and Technology, Rolla, MO 65409, USA; akl5f9@umsystem.edu (A.K.V.); egomaa@umsystem.edu (E.G.); elgawadym@umsystem.edu (M.A.E.); 2 Komar University of Science and Technology, Sulaymaniyah 46001, Iraq

**Keywords:** controlled low-strength material, hazardous waste solidification, off-specification fly ash, bottom ash, geopolymer, flowability, industrial by-product recycling

## Abstract

Conventional controlled low-strength material (CLSM) is a self-consolidating cementitious material with high flowability and low strength, traditionally composed of cement, sand, and water. This study explores the sustainable utilization of off-specification fly ash (OSFA) and bottom ash (BA), classified as industrial by-products with potential environmental hazards, to develop eco-friendly geopolymer CLSM as an alternative to conventional CLSM. Sodium hydroxide (NaOH) was used as an alkali activator to stabilize and solidify both two-part (liquid NaOH) and one-part (solid NaOH pellets) geopolymer CLSM mixtures. These mixtures were evaluated based on flowability (ASTM D6103-17) and compressive strength (<300 psi per ACI Committee 229 guidelines for excavatability). A cost analysis was also conducted. The results demonstrated that incorporating OSFA as a cement replacement increased water demand by 15% to meet flowability requirements, while BA substitution for sand led to segregation challenges requiring mixture adjustments. For two-part mixtures, higher carbon content in OSFA necessitated an increased water-to-fly ash ratio. All self-consolidating mixtures exhibited 1-day compressive strengths ranging from 5 psi (0.03 MPa) to 87 psi (0.6 MPa). One-part mixtures showed a 1% to 34% reduction in 7-day compressive strength compared to two-part mixtures, improving excavatability. Increasing the BA-to-OSFA ratio from 1:1 to 3:1 reduced water demand due to lower surface area but increased the NaOH/OSFA ratio. This study highlights the potential of geopolymer CLSM to reduce costs by up to 94% at current NaOH prices (USD 6 per cubic yard) while repurposing hazardous industrial by-products, offering a cost-efficient, sustainable, and environmentally responsible solution for CLSM production.

## 1. Introduction

Controlled low-strength material (CLSM), as defined in the ACI Committee 229 Report [1], is a self-consolidating cementitious material used in applications where high flowability and low strength are required. The material can be used as an alternative to compacted fills for challenging placement operations, for it does not require vibration or compaction equipment. Common uses for CLSM include backfilling applications, such as structural fills, conduit trenches, and pipe beddings [1]. The primary components for this material are fine aggregate, water, and cement. Other additives, such as supplementary cementitious materials and admixtures, have also been incorporated to improve certain properties [1,2,3,4,5,6]. Given the potential for hazardous by-products like off-specification fly ash (OSFA) and bottom ash (BA) to pose risks to the environment, their utilization in CLSM production represents a sustainable waste management strategy. By repurposing these industrial by-products, CLSM can mitigate the environmental hazards associated with their disposal while reducing reliance on cement. A quality control program is in place for this material to contain specifications for the plastic characteristics, including flowability, consistency, and unit weight and hardened characteristics, including strength, durability, and permeability [1].

Flowability, a key plastic property tested to control the self-consolidating properties, is tested per the “Spread Test” according to ASTM D6103 [7]. This test measures the diameter of the spread of the material, and it is required to have a spread of at least 8 inches (203 mm) without segregation. For infrastructure-related applications, the CLSM must be excavatable to allow embedded infrastructure to be repaired or replaced as needed. Therefore, the compressive strength of the material is limited by this parameter. If hand tools are to be used for excavation, the compressive strength is limited to 100 psi (0.7 MPa). This limit is increased to 300 psi (2.07 MPa) if machinery is used for excavation. If the CLSM is used as a structural fill, however, the compressive strength can be up to 1200 psi (8.27 MPa) [1,3]. Other quality control testing can be performed per ASTM standards on the following properties: compatibility with plastics, excavatability, permeability, potential for corrosion, segregation, setting time, settlement, shrinkage, subsidence, and thermal insulation/conductivity [2,8].

The two primary components, cement and sand, have associated global issues. The cement industry, for instance, is a known contributor to CO_2_ emissions [9], with China being the world’s largest producer and consumer of cement by a significant margin, followed by India, Vietnam, the United States, and Turkey [10]. For every metric ton of cement produced, there is an equivalent metric ton of CO_2_ emitted [11,12]. Additionally, a common fine aggregate for CLSM, sand, is in a global shortage crisis. In 2016, there were over 1 billion metric tons produced, which is a 24% increase as compared to the previous five years [13]. The resulting erosion and sedimentation from the sand mining process have threatened the lives of surrounding aquatic and human life by altering ecosystems and increasing the vulnerability to floods and storms, respectively. Also, due to the increasing demand and shortage in supply, violent conflict has become a rising issue [14]. To reduce the negative impacts caused by cement production and natural resource depletion, material alternatives should be implemented in CLSM.

The coal combustion industry produces 40% of the energy for the globe [15]. Due to this high production, resultant waste products are created at alarming rates. Over 100 million tons of coal combustion products (CCPs) are produced in the U.S.A on an annual basis [16,17]. In 2017, nearly 40% of all CCPs remained unused—14 million tons being unused fly ash (FA) and 5 million tons being unused bottom ash (BA) [17]. Unused FA and BA are disposed of in landfills or ash ponds—lined manmade lakes filled with coal ash [18,19]. Over 100 communities have been subject to health and safety hazards caused by improper lining technologies in the ash ponds [20]. In the event of lining failure, toxic metals from the coal ash materials can leach into the earth, thus contaminating sources of drinking water. This can cause negative health effects such as cancer and nervous system failure [21]. The cost for maintenance and monitoring of the disposal systems, along with the potential liability, can prevent the success of the coal power companies. In addition to the USA, the annual production of CCPs in million tons was 585, 226, 103, and 12.6 in China, India, Europe, and Australia [22].

Partial and full replacements of cement have been performed with alternative materials, such as blast furnace slag powder [23], cement kiln dust [24], cementless binder [25,26], gypsum wallboard [27], spray dryer ash [28], stainless steel reducing slag [29], and wood ash [30,31]. However, most of these studies, due to a reduction in compressive strength, recommended only a partial replacement of cement when using the respective alternative material [23,28,29,30,31].

An alternative to the hydration process, geopolymerization, has also been used throughout research to eliminate the negative effects of cement production. Geopolymerization is the chemical process that occurs between Al- and Si-rich materials and an alkaline activator [32]. The geopolymerization process can be described by the equation below, which is totally different from the common hydration process that takes place in Portland cement.

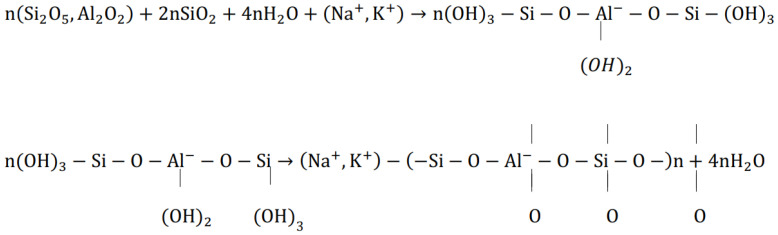



This process has been heavily implemented in concrete by using industrial waste materials, such as FA [23,32,33,34,35,36,37,38,39,40], metakaolin [32,41,42], and slag [38,43]. However, it has been minimally experimented with in the CLSM industry [44,45]. Additionally, partial to full replacements of sand have been performed with artificial aggregate made of bauxite residue [25], BA [28,46,47,48,49], pond ash [25], cinder aggregate [50], crushed glass [28,51], crushed stone powder [52], corrugated fiberboard [53], crumb rubber [28,54,55,56,57], foundry sand [30,58,59], native soil [6,26,60], quarry dust [46], recycled concrete fines [28], treated oil sand waste [61], and waste oyster shells [62]. In these studies, there were observations of reduced compressive strength [6,53,54,56,59,62] and increased water demand [53,54,62], bleeding [53,54,55], and entrapped air [53,54].

BA has been successfully incorporated in CLSM mixture proportions as a fine aggregate replacement [28,44,46,47,48,49]. However, cement was still used [28,46,47,48,49], and replacing it partially was the focus of most of these studies [28,46,47,48,63,64]. Therefore, the associated issues of cement, sand, or coal ash were not eliminated. In one study, full replacements of cement and sand were performed by using geopolymer theory [44], thus addressing all three issues aforementioned. Class F FA, a common material incorporated in CLSM, was used with both lime and sodium hydroxide (NaOH) solution to create the geopolymerization reaction, which has no negative environmental impact after the complete reaction.

By using the geopolymerization mechanism, coal ashes can be used as full replacements for cement and fine aggregate simultaneously in CLSM mixture proportions. This can reduce coal ash waste, greenhouse gas emissions from cement production, and negative effects from natural resource depletion. In addition, the cost of CLSM can be significantly reduced, specifically in countries that rely on the coal combustion industry or use coal as a source of energy, which leads to producing off-specification fly ash and bottom ash, such as, but not limited to, China, India, the USA, the EU, and Australia.

This paper investigates the feasibility of fully replacing cement and/or fine aggregate in CLSM with off-specification fly ash (OSFA) and BA, respectively, through geopolymerization. First, three reference mixtures were made: a conventional CLSM, a CLSM with the cement fully replaced with OSFA and SH, and a CLSM with the sand fully replaced with BA. Then, geopolymer mixtures were made by simultaneously performing a full replacement of cement with OSFA and NaOH and a full replacement of sand with BA. The study analyzed water-, SH-, and BA-to-OSFA ratios to optimize the compressive strength parameters while meeting flowability and excavatability specifications. Two-part mixtures using 10M NaOH solution and one-part mixtures using dry NaOH pellets were compared. Additionally, this study performed a cost analysis on the geopolymer CLSM in reference to conventional CLSM.

## 2. Material Characterization and Properties

Two types of each OSFA and BA were used in this study. Each of these types of coal ash was sourced from two coal power plants: the Labadie power plant of Labadie, Missouri, and the Meramec power plant of St. Louis, Missouri.

### 2.1. Off-Specification Fly Ash

**Figure 1:**
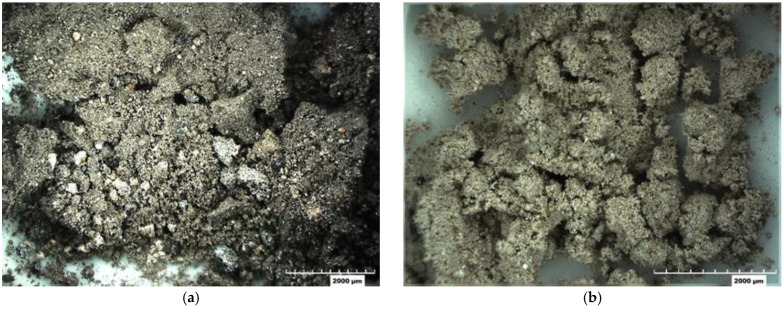
Off-specification fly ash: (**a**) Meramec (F9) and (**b**) Labadie (F1).

The OSFA was received in a dry condition. Figure 1 shows images of the Meramec (F9) and Labadie (F1) materials, named based on the carbon contents.

A chemical analysis was performed on the OSFA using X-ray fluorescence (XRF) to determine the mineral composition, and the loss on ignition (LOI) was determined per ASTM D7348 [65]. Table 1 shows the chemical composition and carbon content, or LOI, by percent weight (%Wt) for the OSFA samples. The values were compared against ASTM C618 [66] for FA classification. The Meramec FA (F9) was determined to be off-specification due to the exceeding carbon content.

### 2.2. Bottom Ash

**Figure 2:**
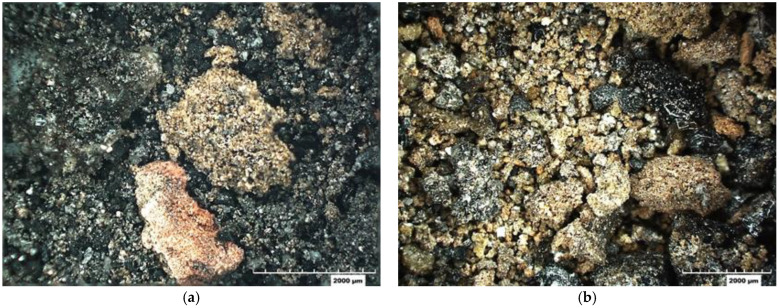
Bottom ash: (**a**) Meramec (B14) and (**b**) Labadie (B2).

The BA from both sources was received in a damp condition. The material was oven-dried at the standard drying temperature of 230 °F prior to experimental usage. Figure 2 shows images of the Meramec (B14) and Labadie (B2) BA, named based on the average carbon contents of the particles.

A chemical analysis using XRF was performed on the powder and fine/coarse BA particles independently (Table 1). The X-ray fluorescence spectroscopy (XRF) was conducted per ASTM D4326-13 [67] using the Oxford X-Supreme 8000 X-ray Fluorescence Spectrometer (Oxford Instruments, Abingdon, UK). The instrument has a capability of energy dispersive X-ray fluorescence to characterize elements Na (11) through U (92). The X-ray Fluorescence (XRF) analysis is a non-destructive technique of determining the elemental composition of materials. The mechanism of the analysis starts after the sampler preparation by irradiating a sample with high-energy X-rays, causing atoms within the sample to emit characteristic fluorescent X-rays. By analyzing the energies and intensities of these emitted X-rays, the elemental makeup of the sample can be identified and quantified. The fine/coarse particles were ground prior to testing. The LOI was also determined, per ASTM D7348, for the BA. Representative samples containing all particle types were ground and mixed thoroughly prior to testing.

**Figure 3:**
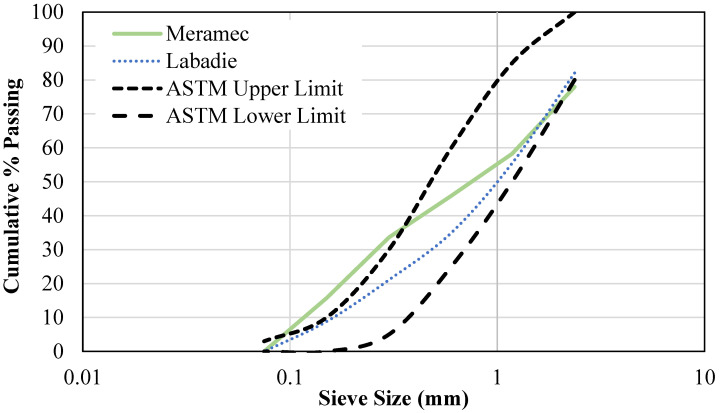
Gradation of bottom ash fine particles.

The physical composition for the BA (Table 2) was determined by a sieve analysis per ASTM C136 [68]: powder particles passing sieve #200 (0.074 mm), fine particles passing sieve #4 (4.76 mm) and retained on #200 (0.074 mm), and coarse particles being retained on sieve #4 (4.76 mm). The gradation of the fine particles (Figure 3) was determined per ASTM C33 [69]. Additional physical properties of the BA fine particles are listed in Table 2.

### 2.3. Activator (Sodium Hydroxide)

NaOH pellets were used as the alkaline activator for the geopolymerization process. For two-part mixture proportions, a liquid NaOH solution was used. For solution preparation, dry NaOH pellets were combined with deionized water to create a 10M NaOH solution. The solution rested for a minimum of two hours prior to usage. For one-part mixture proportions, no preparation was required, and dry NaOH pellets were used.

## 3. Experimental Program

### 3.1. Test Matrix

Thirty-eight unique mixture proportions were studied. The test matrix is separated by color per material source used: conventional materials, Meramec coal ash(es), and Labadie coal ash(es). The mixtures are organized by increasing NaOH content and then by increasing water content. The proportions by weight for the following materials are listed in Table 3: cement (C), 10 M NaOH Solution (NS), NaOH pellets, class C FA, OSFA, river sand (RS), BA, and water (W).

The mixture proportions labels contain three terms. The first term is the test type for the mixture proportions: reference mixtures (R), two-part mixtures (2P) using liquid 10 M NaOH solution, one-part mixtures (1P) using dry NaOH pellets, and one-part ratio-adjusted mixtures (1PA) using dry NaOH pellets with an increased BA/OSFA ratio. The second term lists the cementitious material and then the aggregate used within the mix. The selection of cementitious materials used includes Portland cement (OPC), Meramec OSFA (F9), and Labadie OSFA (F1). The selection of aggregates used includes RS, Meramec BA (B14), and Labadie BA (B2). The third term listed for the two-part mixtures specifies the NaOH solution content. The third term for the one-part and one-part ratio adjusted mixtures was made to match the respective two-part equivalent mix. Mixtures with the same NaOH content contain letter suffixes, as needed, where “a” has the lowest water content and “d” has the highest water content. For instance, “2P-F9/B14-20a” is the two-part (2P) mixture that uses FA with a carbon content of 9% (F9) as the cementitious material and BA with a carbon content of 14% (B14) as the aggregate. The mixture has a 10 M NaOH solution-to-OSFA ratio of 0.20, and it has the lowest water content (a) for all mixtures having the same materials and NaOH content.

Reference (R) mixtures were made to analyze the OSFA and BA substitutions independently. A conventional ordinary Portland cement mixture (R-OPC/RS) using cement and RS was made for reference. Full cement replacement mixtures were made by using the respective OSFA and NaOH pellets as full replacements for cement and RS as the aggregate: R-F9/RS and R-F1/RS. Then, full aggregate replacement mixtures were made by using cement and using the respective BA as a full replacement for sand: R-OPC/B14 and R-OPC/B2.

For the next test matrix, two-part (2P) geopolymer CLSM was made by performing full cement and aggregate replacements simultaneously. OSFA and 10 M NaOH solution were used as the cement replacement, and BA was used as the aggregate replacement. The two-part mixture proportions maintained a BA-to-OSFA ratio of 1:1 for this analysis. The NS and W contents were the only parameters changed.

One-part (1P) mixture proportions were then developed to compare with the equivalent two-part mixture proportions. To improve the user-friendliness of the material, the need for corrosive solution handling was eliminated. Instead of preparing a 10 M NaOH solution, the dry NaOH pellets were added directly into the mixer, along with the coal ashes, so only water needed to be added throughout the mixing process. The equivalent amount of water used to make the NaOH solution was added into the mixing water. Therefore, the net content of NaOH pellets and water used in the two-part and one-part mixture designs remained consistent.

Lastly, one-part ratio adjusted (1PA) designs were developed to analyze the effects of increased BA/OSFA content. The one-part mixtures have a 1:1 BA/OSFA, whereas the 1PA mixtures have a 3:1 BA/OSFA. The other parameters, such as NaOH and water, remained equivalent to the one-part mixture counterpart.

All mixtures were prepared by adding dry materials first and then adding liquid component(s). The mixture proportions were tested by performing flowability and compressive strength tests and cost analyses.

### 3.2. Flow Consistency Test

**Figure 4:**
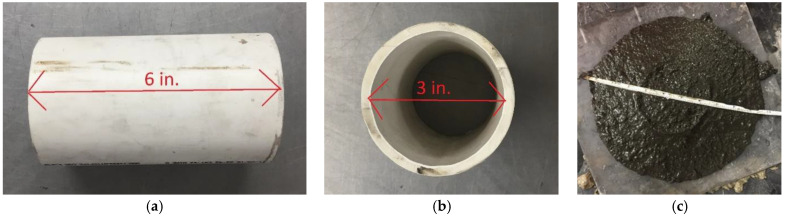
Spread test: (**a**) apparatus length, (**b**) apparatus diameter, (**c**) measurement.

The flowability of the mixtures was tested by the flow consistency method, as specified by ASTM D6103. The test is performed by filling a hollow cylinder (Figure 4a,b) with CLSM, vertically lifting the cylinder, and measuring the spread diameter of the CLSM (Figure 4c). To have CLSM of proper consistency, the spread should be at least 8 inches (203 mm) without segregation per ASTM D6103 [7].

### 3.3. Compressive Strength Test

The CLSM mixtures were placed into wood cube molds. Cylinder molds were not used for material placement since the low strength of the material would inhibit the ability to demold the CLSM without damaging the specimens. The reference mixtures (R) were placed in 3.5″ cubes, and the remaining mixtures (2P, 1P, and 1PA) were placed in 5.5″ cubes.

The molds were placed in an ambient environment directly after material placement. The specimens were then demolded after 24 h of curing. After the designated curing time, the cube specimens were tested. The load was applied at 200 pounds per minute until specimen failure. Since the specimens were placed in cube molds, the compressive strength results of 3.5″ and 5.5″ cubes were divided by the appropriate factors of 1.08 and 1.09, respectively, to account for shape variability.

### 3.4. Cost Analysis

The cost was determined for the one-part and two-part geopolymer mixtures that met flowability and compressive strength specifications per ASTM D6103 [7] and ACI Committee 229 [1]. The costs of the OSFA, BA, and water were neglected. Only the cost of NaOH pellets was used to calculate the cost of the geopolymer CLSM mixtures. The maximum and minimum prices for the NaOH pellets were designated as USD 0.15/lb and USD 0.45/lb per the 2018 annual cost report by InfoMine USA [75]. A reference cost for conventional CLSM was used per Rolla Ready Mix prices [76].

## 4. Results and Discussion

### 4.1. Flow Consistency

#### 4.1.1. Reference Mixtures

The reference cement mixture (R-OPC/RS) and cement replacement mixtures containing OSFA and NaOH pellets, R-F9/RS and R-F1/RS, were able to achieve the minimum spread of 8″, 8″, and 9″, respectively, without segregation. However, the aggregate replacement mixtures containing BA, R-OPC/B14 and R-OPC/B2 achieved spreads of 8″ and 5″ and did not meet the flowability specifications due to segregation.

**Figure 5:**
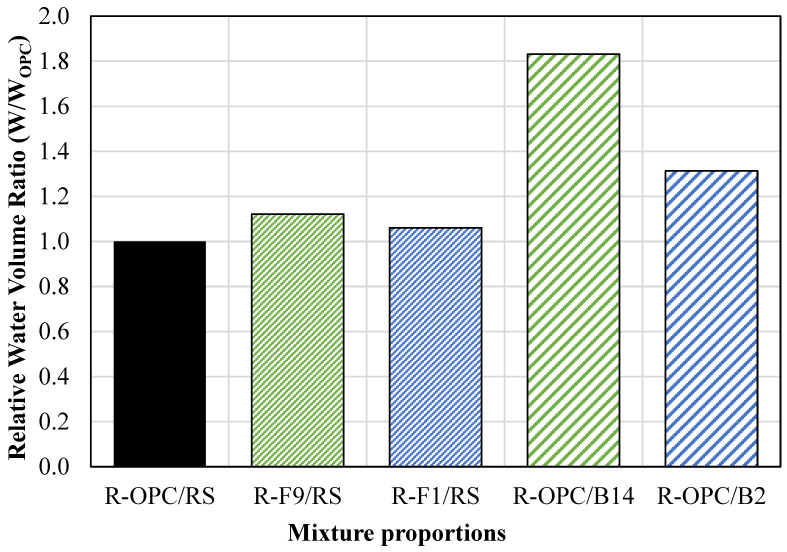
Water volume ratio for single constituent replacement.

The single constituent replacement mixtures were tested using the equivalent volume of water as the conventional CLSM mixture (R-OPC/RS). However, additional water was needed to achieve flowability. The ratio of water used (W) to water used in conventional CLSM (W_OPC_) was determined. The water volume ratio (W/W_OPC_) for each reference mixture is displayed in Figure 5. The cement replacement mixtures required approximately 15% more water to achieve the required flowability for self-consolidation. The aggregate replacement mixtures also required additional water. The R-OPC/B14 mixture required 80% more water to achieve an 8″ spread. However, this mixture did not meet flowability specifications due to segregation. Additionally, the R-OPC/B2 mixture required 30% more water to have a 5″ spread and was already subject to segregation. Therefore, this mixture also did not meet flowability requirements. The high-water demand and segregation effects were caused by the high content of powder within the BA. However, the mixture proportions could be adjusted to reduce these issues.

#### 4.1.2. Two-Part Mixtures

**Figure 6:**
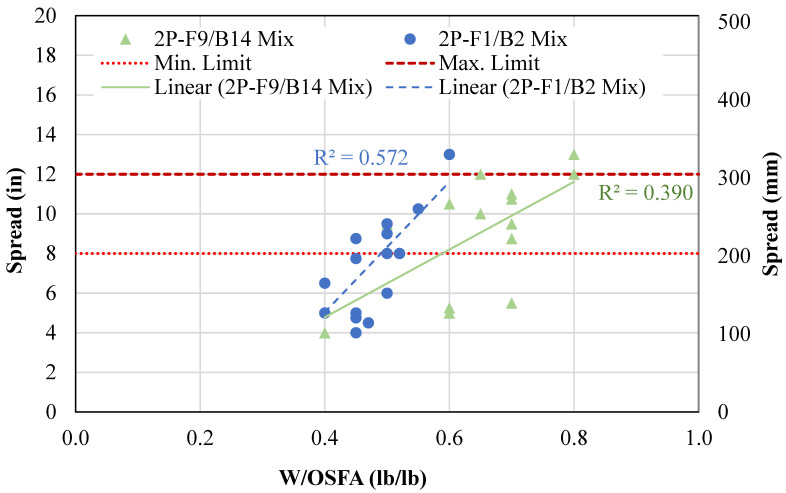
Water content vs. flowability for two-part mixtures.

A geopolymer CLSM was then developed by fully replacing both cement and sand with OSFA and NaOH solution and BA, respectively. Figure 6 shows the relationship between increasing water content and flowability, along with the minimum limit set by ASTM D6103 [7] and the maximum limit for no segregation. Water content is represented as the water-to-OSFA ratio by weight (W/OSFA), and flowability is represented as spread. The spread was increased by increasing the water content. Mixtures containing FA9 and FA1 required W/OSFA 0.6 and 0.5, respectively, to meet the minimum spread limit. Excess water led to segregation and bleed water, primarily in mixtures with spread measuring above 12 inches (305 mm). The mixtures containing F9 and B14 had segregation at a W/OSFA of 0.8, and mixtures containing F1 and B2 had segregation at a W/OSFA of 0.6. The mixtures containing F9/B14 required more water for flowability due to the higher powder composition in the BA.

**Figure 7:**
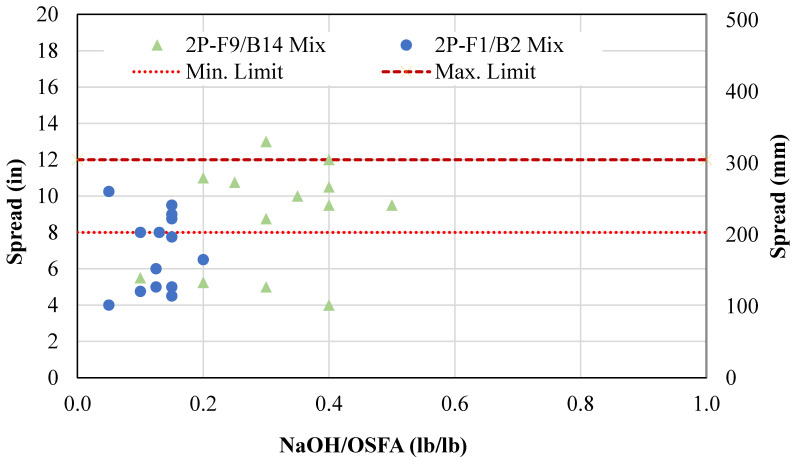
NaOH solution vs. flowability for two-part mixtures.

Figure 7 shows the relationship between NaOH/OSFA and spread. These two factors did not show a correlation. This is likely due to the combatting mechanisms of water and alkaline activator with the coal ashes. The NaOH solution, due to being in liquid form, was able to coat the material and aid in flowability. However, the alkaline content of the solution reacted with the coal ash particles in the mixture upon contact. This reaction caused a stiffening reaction as the geopolymerization and hydration processes were activated. The water content of the solution promoted flowability of the mixture; however, the NaOH content accelerated the setting of the material. Therefore, the solution did not provide a direct correlation with flowability.

#### 4.1.3. One-Part Mixtures

**Figure 8:**
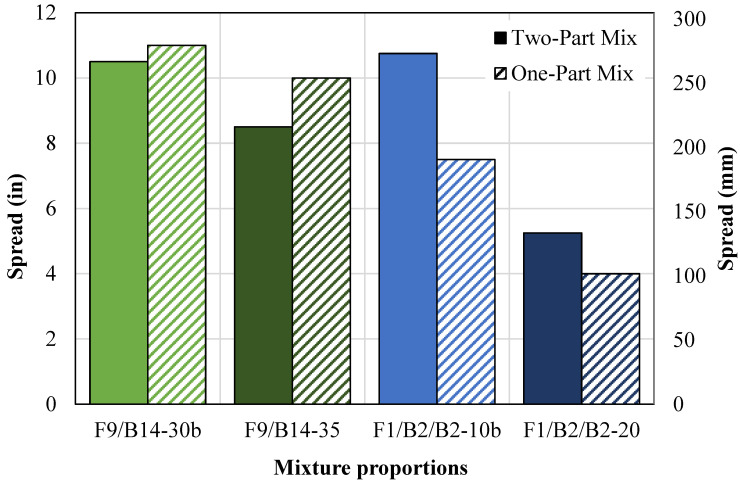
Two-part vs. one-part mixture flowability.

The flowability results for the one-part mixtures and equivalent two-part are shown in Figure 8. Two mixtures of each coal ash were selected, with one containing a low NaOH content and one containing a high NaOH content. For the one-part mixture, the NaOH pellets were added directly to the mixer, thus making the mixing procedure highly exothermic. Additionally, the water influenced the flowability since the equivalent water used to make the NaOH solution in the two-part mixture was instead added to the mixing water in the one-part mix.

The 1P-F9/B14-30b mixture had a 0.09 NaOH pellet ratio and a 0.91 water ratio, and the 1P-F9/B14-35 mixture had a 0.10 NaOH pellet ratio and a 0.90 water ratio. Both one-part mixtures had increases in spread (Figure 8). The 1P-F9/B14-30b mixture had a spread 5% greater than the two-part mixture, and the 1P-F9/B14-35 had a spread 18% greater than the two-part mixture. Both one-part mixtures showed insignificant effects caused by the increase in heat during mixing, so the increase in water used for mixing governed the change in flowability. The ratio of one-part versus two-part mixture spread, represented as 1.05 and 1.18, in relationship to the equivalent NaOH solution content is consistent between both mixtures, thus confirming that the spread is directly proportional to the additional mixing water.

The 1P-F1/B2-10b mixture had a 0.03 NaOH pellet ratio and a 0.57 water ratio, and the 1P-F1/B2-20 mixture had a 0.06 NaOH pellet ratio and a 0.54 water ratio. Both one-part mixtures had decreases in spread (Figure 8). The 1P-F1/B2-10b mixture had a spread 30% less than the two-part mix, and the 1P-F1/B2-20 mixture had a spread 24% less than the two-part mix. The exothermic reaction during mixing, along with the lower W/OSFA, caused an acceleration in the reaction rate. This produced earlier stiffening of the material in the one-part mixtures, thus reducing the spread.

#### 4.1.4. One-Part Ratio-Adjusted Mixtures

**Figure 9:**
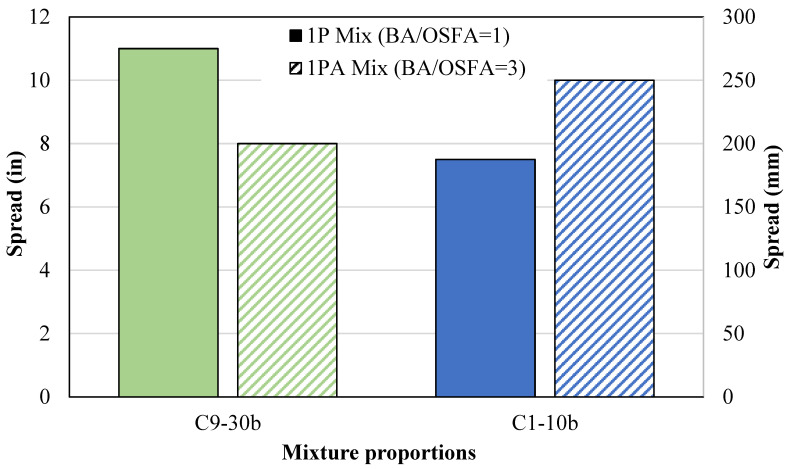
One-part vs. one-part ratio-adjusted mixture flowability.

One optimum one-part mixture design from each source was selected to analyze the effects of increasing BA content. In Figure 9, the flowability of the one-part ratio-adjusted mixtures (1PA-F9/B14 and 1PA-F1/B2) containing a higher BA/OSFA ratio of 3:1 is compared to the equivalent one-part mixtures with a BA/OSFA ratio of 1:1. Per the figure, the one-part ratio-adjusted mixture containing F9 and B14 (1PA-F9/B14) decreased in flowability due to the increase in the NaOH/OSFA ratio. The increased geopolymerization reaction accelerated the stiffening of the material. Despite these occurrences, the flowability of the mixture with the increased BA/OSFA ratio (1PA-F9/B14-30b) still maintained the required flowability for self-consolidation. Additionally, the one-part ratio-adjusted mixtures containing F1 and B2 (1PA-F1/B2-10b) increased in flowability due to the increase in BA content. The decrease in surface area due to the larger particle sizes of the BA reduced the amount of water needed to achieve the required flowability.

### 4.2. Compressive Strength

**Figure 10:**
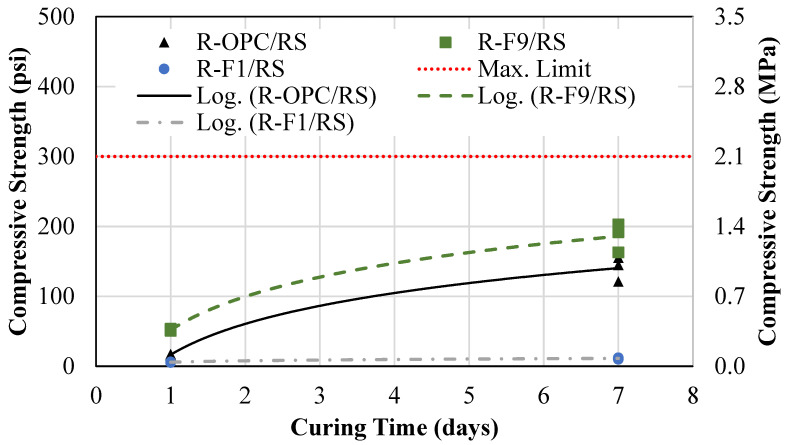
Conventional vs. cement replacement mixture compressive strength.

A cement volume replacement was performed by replacing it with OSFA and NaOH pellets. Figure 10 shows the results of the 1-day and 7-day compressive strength for the conventional CLSM mixture (R-OPC/RS), the cement replacement mixture containing FA with a 9% carbon content (R-F9/RS), and the cement replacement mixture containing FA with a 1% carbon content (R-F1/RS). The average 1-day and 7-day compressive strengths for the R-OPC/RS mixture were 16 psi (0.11 MPa) and 202 psi (1.39 MPa). For the R-F9/RS mix, the average 1-day and 7-day compressive strengths were 52 psi (0.36 MPa) and 186 psi (1.28 MPa). For the R-F1/RS mix, the average 1-day and 7-day compressive strengths were 6 psi (0.04 MPa) and 11 psi (0.08 MPa).

The R-F9/RS mixture showed comparable strength to the R-OPC/RS mix; however, it was approaching the excavatability limit. Therefore, the NaOH content must be reduced if the material is to be used for applications requiring excavation. The R-F1/RS mix, on the other hand, is significantly lower than the R-OPC/RS mix, thus meeting excavatability requirements. However, the NaOH content must be significantly increased if the material is to be used for structural fill applications.

**Figure 11:**
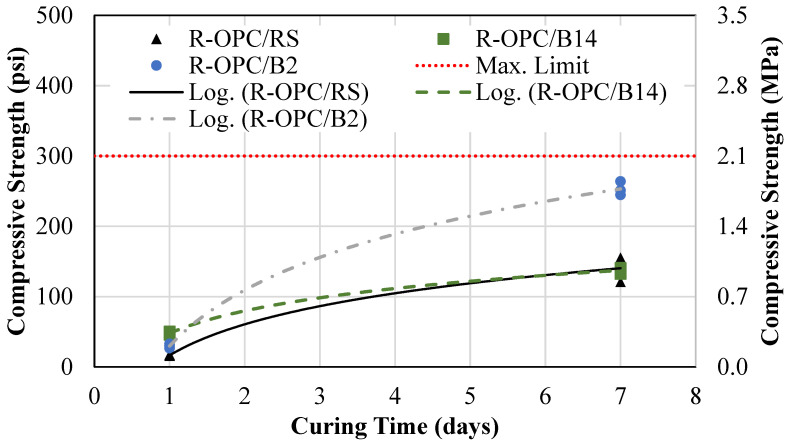
Conventional vs. aggregate replacement mixture compressive strength.

An aggregate volume replacement was performed by replacing the RS with BA. Figure 11 shows the results of the 1-day and 7-day compressive strength for the conventional CLSM mixture (R-OPC/RS), the aggregate replacement mixture containing BA with a 14% carbon content (R-OPC/B14), and the aggregate replacement mixture containing BA with a 2% carbon content (R-OPC/B2). The average 1-day and 7-day compressive strengths for the R-OPC/RS mixture were 16 psi (0.11 MPa) and 202 psi (1.39 MPa). For the R-OPC/B14 mix, the average 1-day and 7-day compressive strengths were 48 psi (0.33 MPa) and 137 psi (0.94 MPa). For the R-OPC/B2 mix, the average 1-day and 7-day compressive strengths were 30 psi (0.21 MPa) and 253 psi (1.74 MPa).

Per the figure, the R-OPC/B14 mixture had nearly equivalent compressive strength results compared to the conventional CLSM. The R-OPC/B2 mix, on the other hand, had a significantly higher 7-day compressive strength, thus suggesting pozzolanic action within the BA.

**Figure 12:**
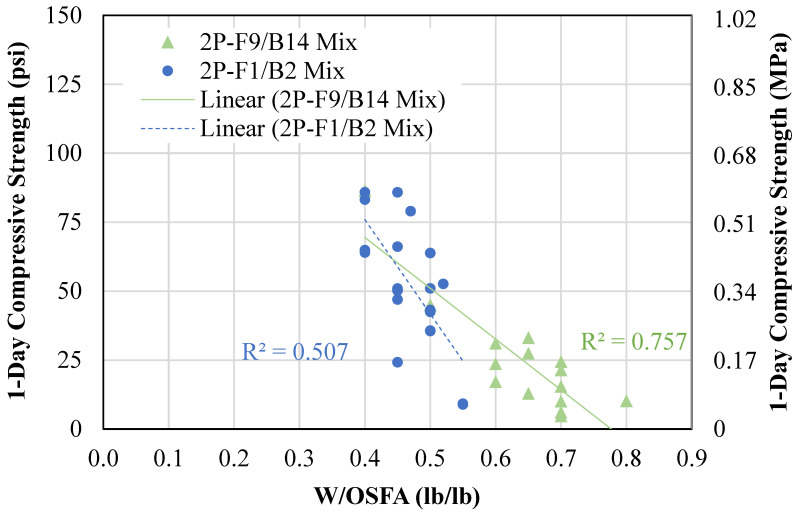
Relationship between water content and compressive strength.

Figure 12 represents the relationship between water content and compressive strength. As the water content increased, the early-age compressive strength decreased. The 2P-F9/B14 mixtures had decreasing 1-day compressive strength from 87 psi (0.60 MPa) to 5 psi (0.03 MPa) for water-to-OSFA ratio (W/OSFA) ranging from 0.4 inches (10 mm) to 0.8 inches (20 mm). The 2P-F1/B2 mixtures had decreasing 1-day compressive strength from 86 psi (0.59 MPa) to 9 psi (0.06 MPa) for W/OSFA ranging from 0.4 inches (10 mm) to 0.6 inches (15 mm). The 2P-F9/B14 mixtures contained BA with a powder composition of 22%, whereas the 2P-F1/B2 mixtures had BA with a powder composition of 9%. The high powder particle composition in the 2P-F9/B14 is the reason for the increased water demand.

The reduction in compressive strength with increasing water content is due to several phenomena, such as weak spots in areas of high free water content and reduction in alkali concentration. The water dilutes the NaOH solution, thereby prohibiting the geopolymerization reaction from occurring. This dilution of alkali solution also decreases the reaction rate, thus significantly extending the setting time and producing low compressive strengths at short cure times.

**Figure 13:**
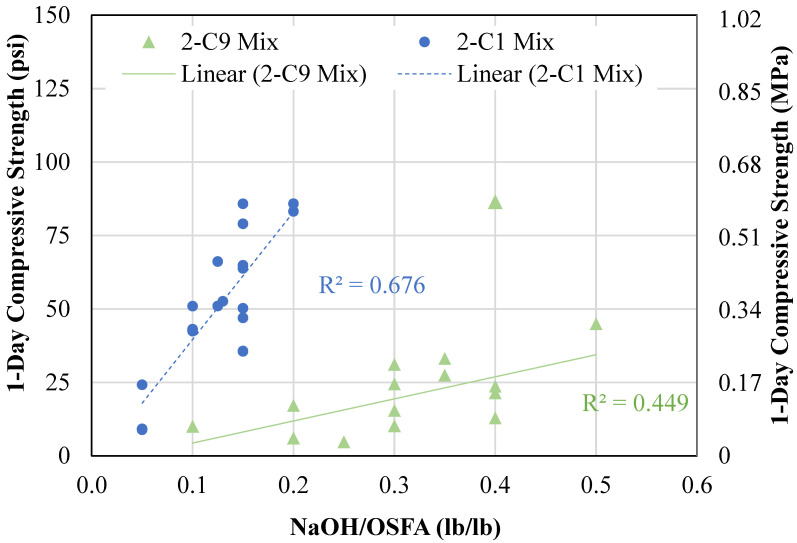
Relationship between NaOH solution content and compressive strength.

Figure 13 shows the relationship between NaOH ratio and early-age compressive strength. As expected, the compressive strength increased as the NaOH content, represented by the NaOH-to-OSFA ratio (NaOH/OSFA), increased. The 2P-F9/B14 mixtures gained in strength from 9 psi (0.06 MPa) to 86 psi (0.59 MPa) as the NaOH/OSFA ratio increased from 0.1 to 0.5. The data point reading 86 psi (0.59 MPa), however, was not included in the trendline since it was considered an outlier. This mixture proportion had a low W/OSFA ratio of 0.4 and had a spread measurement of 4 inches (102 mm). The 2P-F1/B2 mixtures containing had a sharp increase in strength, increasing in 1-day compressive strengths from as low as 5 psi to strengths as high as 87 psi (0.6 MPa) with only an increase in NaOH/OSFA from 0.5 to 0.2. This is due to the accelerated reaction rate caused by the geopolymer mechanisms between the alkali solution and coal ash materials. The higher concentration mixtures—mixtures containing the same water content but increased NaOH solution content—increase the rate of reaction, while the increased volume of solution improves the particle coating capabilities and coal ash-alkali contact.

As mentioned, the 2P-F9/B14 mixtures had a high water content due to the high particle surface area, whereas the 2P-F1/B2 mixtures had a maximum water content of 0.6. Therefore, the lack of water in the 2P-F1/B2 mixtures increased the overall alkaline concentration within the mix, producing a sharper trendline.

**Figure 14:**
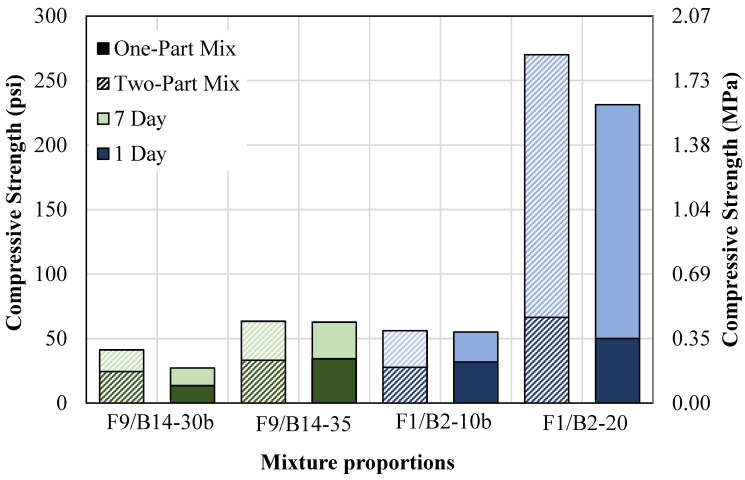
Two-part vs. one-part compressive strength results.

The mechanical properties for the two-part and one-part mixtures were also compared. Figure 14 shows 1-day and 7-day compressive strength results for two-part and one-part mixtures containing F9 and B14 with a low NaOH content (F9/B14-30b) and a high NaOH content (F9/B14-35). Additionally, the figure shows the results for the two-part and one-part mixtures containing F1 and B2 with a low NaOH content (F1/B2-10b) and a high NaOH content (F1/B2-20).

The compressive strengths for the two-part F1/B2-30b were 24 psi (0.17 MPa) and 41 psi (0.28 MPa), and the one-part mixture results were 13 psi (0.09 MPa) and 27 psi (0.19 MPa). The two-part F1/B2-35 mixture compressive strengths were 33 psi (0.23 MPa) and 63 psi (0.43 MPa), and the one-part mixture results were 34 psi (0.23 MPa) and 63 psi (0.43 MPa). The figure shows that the low NaOH mixture (F1/B2-30b) had a decrease in compressive strength for all cure durations of one-part mixtures compared to the two-part mixtures. This result is caused by the increased amount of water used directly for mixing. Due to the potential non-homogeneity of NaOH within the mixture, high water content in particular regions could effectively dilute the NaOH, thereby creating weak spots and reducing the compressive strength. However, this reduction in strength is an advantageous property for CLSM, for a decelerated strength development can improve the excavatability of the material. As seen for the high NaOH mixture (F1/B2-35), however, the compressive strengths for the two-part and one-part mixtures are the same. Therefore, mixtures containing low NaOH are more susceptible to the increased water due to the lack of strengthening mechanisms.

The compressive strengths for the two-part F1/B2-10b mixture were 27 psi (0.19 MPa) and 56 psi (0.39 MPa), and the one-part results were 32 psi (0.22 MPa) and 55 psi (0.38 MPa). The two-part F1/B2-20 mix 1-day and 7-day compressive strengths were 66 psi (0.46 MPa) and 270 psi (1.86 MPa), and the one-part mix results were 50 (0.34 MPa) and 231 psi (1.59 MPa). Therefore, the mixing method, whether using NaOH solution or dry pellets, does not significantly affect the early-age strength up to 7 days.

**Figure 15:**
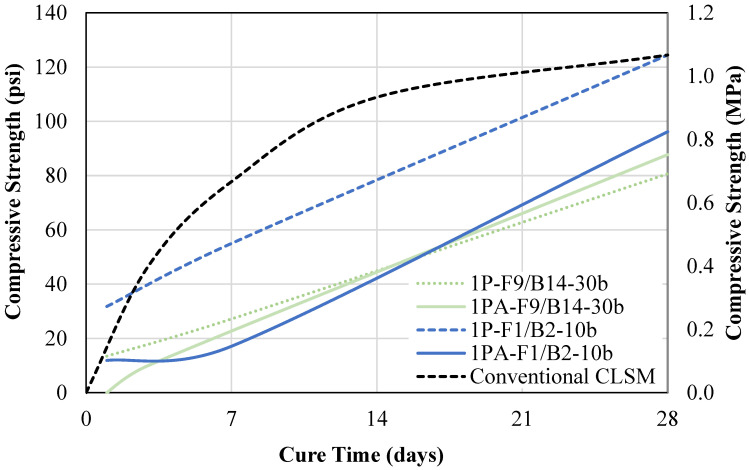
One-part vs. one-part ratio adjusted compressive strength.

The one-part mixtures were then altered to increase the BA content. The one-part (1P) mixtures have a 1:1 BA-to-OSFA ratio, whereas the one-part ratio adjusted (1PA) mixtures have a 3:1 ratio. The compressive strengths of the ratio-adjusted mixtures were compared alongside the one-part mix equivalents. Figure 15 represents the results for the F1/B2-30b mixtures. The 1PA-F9/B14-30b specimens were unable to be demolded at day 1. However, the strength increased significantly to 73 psi (0.5 MPa), surpassing the strength of the one-part mix, by day 28. The reduction in 1-day strength was primarily caused by the limitation in strength by the BA fine/coarse particles. Since these particles are inert, only the powder particles can contribute to the strength development mechanism. Therefore, the increase in inert material resulted in a decrease in early-age strength. However, over time, the strength development rate increased as the NaOH and water particles continued to react with the powder particles.

Additionally, Figure 15 compares the compressive strengths of the one-part and ratio-adjusted equivalents for the F1/B2-10b mix. A theoretical conventional CLSM strength development curve is also displayed to show how the 1P-F1/B2-10b curve would appear if he had the same behavior as conventional CLSM. The mix with increased BA content, 1PA-F1/B2-10b, had compressive strengths of 12 psi (0.08 MPa), 17 psi (0.12 MPa), and 96 psi (0.66 MPa) at 1, 7, and 28 days. The compressive strength is significantly lower than the one-part mix equivalent for all testing dates. This is due to the increase in inert material and decrease in OSFA. However, this can be advantageous, as it improves the excavatability of the material.

### 4.3. Cost Analysis

The cost for geopolymer CLSM was compared to current CLSM market prices to determine the economic feasibility of the new material. The cost for conventional CLSM is approximated at USD 90 per cubic yard [76], but this price can vary by location, materials, and application. The geopolymer materials contain four key components: BA, OSFA, NaOH pellets, and water. Maintenance, storage, and disposal of coal ash waste can be significant expenses to coal power companies. Therefore, the costs for these off-specification coal ashes are nonexistent, for coal plants will distribute this material for free as to eliminate the need for permanent material handling. Then, NAOH pellets, depending on the manufacturer and purity of the chemical, can range in cost from USD 0.15/lb to USD 0.45/lb [75]. Water and transportation were neglected for this analysis due to the consistency between materials, along with the assumption that if industrialized, local ready mixtures would carry these materials in addition to conventional CLSM components. Therefore, the cost for geopolymer CLSM is solely reliant on the content of NaOH. However, by looking at the weight proportions in Table 3, it can be noticed that the water weight proportions for conventional CLSM with Portland cement are between 0.95 and 1.25, while they are between 0.45 and 0.7 for the proposed geopolymer CLSM in Table 4. That shows a saving of almost half of the water cost when geopolymer CLSM is used.

**Figure 16:**
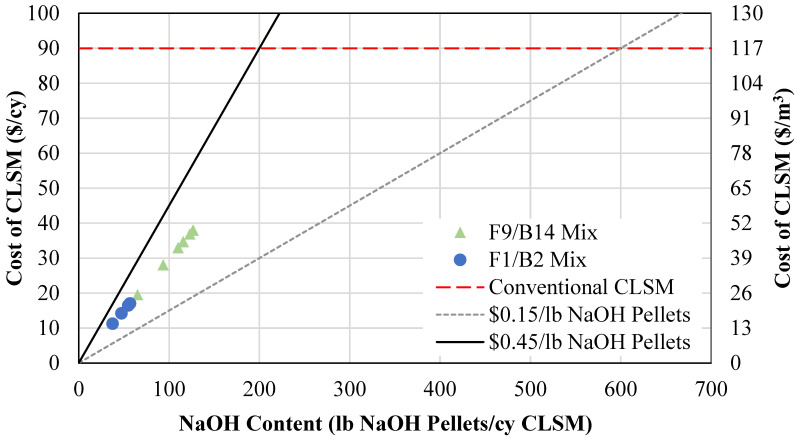
Geopolymer vs. conventional CLSM cost analysis.

Figure 16 illustrates the economic feasibility of using geopolymer CLSM based on the cost of NaOH. A low-cost range of USD 0.15/lb of NaOH and a high-cost range of USD 0.45/lb of NaOH are plotted on a graph with respect to pounds of NaOH pellets. These lines were then used to determine the amount of NaOH pellets that can be used per cubic yard (cy) of CLSM to be economically feasible compared to conventional CLSM. From this figure, the geopolymer mixture proportions are economically advantageous as long as the content of NaOH pellets stays between 200 and 600 pounds per cubic yard of CLSM for the maximum and minimum prices of NaOH, respectively. These optimized mixtures meeting flowability and compressive strength requirements, listed in Table 4, were further analyzed to determine the average cost for these mixture proportions per the market value of NaOH. These values are plotted in Figure 16 to show that all optimized mixtures meet the requirement for economic feasibility.

Table 4 shows the cost comparison for the conventional CLSM mixtures with the geopolymer mixtures. The mixtures meeting flowability and compressive strength requirements are listed, along with the respective costs per cubic yard: minimum (cost, min), maximum (cost, max), and average (cost, ave). The minimum, maximum, and average costs are USD 0.15, USD 0.45, and USD 0.30 per lb of NaOH. Per this table, the geopolymer CLSM mixtures have considerably lower costs than that of conventional CLSM, saving up to 94% of total material costs. These costs can be further reduced if fewer NaOH pellets are used to improve excavatability.

## 5. Conclusions

Presented in this study is a sustainable alternative to conventional CLSM by performing a full replacement of cement and sand. Thirty-eight mixtures using two sources, OSFA and BA, were tested. The BA was used as the fine aggregate, and OSFA and NaOH were used as the binding agents to develop geopolymer CLSM. The proportions of water, NaOH, and BA were varied within the mixtures. The effects of NaOH, being in a 10M solution form in the two-part mixtures and in a dry pellet form in the one-part mixtures, were tested additionally. Flowability and compressive strength tests were performed to create mixture proportions that met specifications set by ASTM [7] and ACI [1]. while a cost analysis highlighted the economic benefits of this approach. This study demonstrates the potential of geopolymer CLSM to reduce environmental hazards associated with OSFA and BA disposal while providing a cost-effective and high-performing construction material. The main findings are as follows:

- An eco-friendly alternative CLSM was developed using off-specification fly ash (OSFA) and sodium hydroxide (NaOH) as a 100% replacement for cement, with bottom ash (BA) serving as the aggregate. This approach not only repurposes hazardous industrial by-products but also contributes to reducing the environmental impact of their disposal. The developed mixtures required 15% more water to meet flowability requirements.
- An alternative CLSM was developed by using cement and BA as a 100% sand replacement. These mixtures were subject to segregation; therefore, adjustments in the mixture proportions must be made to meet flowability requirements.
- A two-part geopolymer CLSM was developed by fully replacing both the cement and sand with OSFA and BA, respectively, and NaOH solution. With the BA-to-OSFA ratio equal to 1, the mixtures containing FA9 required a W/OSFA ratio between 0.6 and 0.8, and the mixtures containing FA1 required a W/OSFA ratio between 0.5 and 0.6 to meet flowability requirements. The mixtures meeting self-consolidation specifications had 1-day compressive strengths measuring between 5 psi (0.03 MPa) and 87 psi (0.6 MPa).
- A one-part geopolymer CLSM, using dry NaOH pellets, was developed. The mixtures containing FA9 increased in spread by at least 5% due to the excess mixing water, but the mixtures containing FA1 decreased in spread by more than 20% due to the heat caused by the exothermic reaction during mixing. The 7-day compressive strength for all one-part mixtures decreased by 1% to 34%, thereby improving the excavatability.
- An increase in the BA-to-OSFA ratio from 1:1 to 3:1 decreased the water demand due to the reduced surface area but increased the NaOH/OSFA ratio. The F9/B14 mixtures showed a decrease in spread by 28% but still met the minimum flowability requirement, and the F1/B2 mixtures showed an increase in spread by 33%. The F9/B14 mixtures increased in compressive strength after 7 days due to the increase in NaOH/OSFA but were still only 30% of the maximum capacity for excavatability by day 28.
- The one-part ratio-adjusted mixtures with F1/B2 had improved excavatability due to the inert characteristics of the fine/coarse BA particles. This mixture measured at a 28-day compressive strength of 96 psi (0.66 MPa), which was 23% lower than the one-part mix.
- The geopolymer CLSM mixture proportions can reduce the cost for CLSM by up to 94% with the current NaOH prices, at a price of USD 6 per cubic yard. Based on the cost analysis, one- and two-part mixtures (F1/B2-10b) with an NaOH solution of 0.10 were the most recommended option.

## 6. Future Work

- It is recommended to evaluate the social impact of the reduction in opportunities in the cement industry as a result of introducing no-cost byproduct as an effective replacement.
- The water absorption and the porosity of the newly proposed geopolymer CLSM need to be investigated for different types of geopolymer CLSM mixtures.
- The long-term performance and durability of the newly proposed geopolymer CLSM need to be investigated.

## Figures and Tables

**Table 1:** Chemical composition (%Wt) of coal ash types.

	Meramec			Labadie		
	Fly Ash (F9)	Bottom Ash (B14)		Fly Ash (F1)	Bottom Ash (B2)	
	Powder	Powder	Coarse	Powder	Powder	Coarse
Na_2_O	1.29	0.39	0.51	1.43	0.61	0.69
MgO	4.99	3.99	3.68	5.36	4.42	3.88
Al_2_O_3_	20.1	17.6	18.1	19.7	18.1	18.3
SiO_2_	38.3	41.6	45.9	36.0	39.9	44.7
P_2_O_5_	0.22	0.20	0.22	1.97	1.11	1.38
K_2_O	0.49	0.49	0.52	0.52	0.44	0.42
CaO	25.0	25.0	21.9	26.9	26.3	23.0
TiO_2_	1.44	1.57	1.48	1.49	1.56	1.44
Fe_2_O_3_	6.54	8.09	8.66	6.65	7.46	6.73
SiO_2_ + Al_2_O_3_ + Fe_2_O_3_	64.9	67.3	70.9	62.4	65.5	69.7
Loss on Ignition	8.54	14.4		0.93	1.70	

**Table 2:** Physical properties of bottom ash.

Standard	Property	B14	B2
ASTM C136 [68]	Powder particle composition, %	21.8	9.20
	Fine particle composition, %	66.7	78.3
	Coarse particle composition, %	11.4	12.4
ASTM C128 [70]	Specific gravity, oven dry	2.53	2.20
	Specific gravity, saturated surface dry	2.58	2.29
	Specific gravity, apparent	2.67	2.41
	Water absorption, %	2.20	4.00
ASTM C117 [71]	Materials finer than #200 sieve, %	2.60	1.17
ASTM D7428 [72]	Micro-Deval, % material lost	12.6	8.48
ASTM C1252 [73]	Uncompacted voids, %	75.2	56.9
ASTM C40 [74]	Organic impurities	0	2

**Table 3:** Mixture proportions matrix by weight proportions.

Test	Mix Design	Cement (C)	NaOH Solution (NL)	NaOH Pellets (NP)	Class C Fly Ash (FA)	Off-Specification Fly Ash (OSFA)	River Sand (RS)	Bottom Ash (BA)	Water (W)
Reference	R-OPC/RS	0.25			0.75		6.75		0.95
	R-F9/RS			0.09		1.00	7.06		1.11 *
	R-F1/RS			0.03		1.00	7.06		1.05 *
	R-OPC/B14	0.25			0.75			2.75	1.74 *
	R-OPC/B2	0.25			0.75			5.48	1.25 *
2-Part	2P-F9/B14-10		0.10			1.00		1.00	0.70
	2P-F9/B14-20a		0.20			1.00		1.00	0.60
	2P-F9/B14-20b		0.20			1.00		1.00	0.70
	2P-F9/B14-25		0.25			1.00		1.00	0.70
	2P-F9/B14-30a		0.30			1.00		1.00	0.60
	2P-F9/B14-30b		0.30			1.00		1.00	0.70
	2P-F9/B14-30c		0.30			1.00		1.00	0.80
	2P-F9/B14-35		0.35			1.00		1.00	0.65
	2P-F9/B14-40a		0.40			1.00		1.00	0.40
	2P-F9/B14-40b		0.40			1.00		1.00	0.60
	2P-F9/B14-40c		0.40			1.00		1.00	0.65
	2P-F9/B14-40d		0.40			1.00		1.00	0.70
	2P-F9/B14-50		0.50			1.00		1.00	0.50
	2P-F1/B2-05a		0.05			1.00		1.00	0.45
	2P-F1/B2-05b		0.05			1.00		1.00	0.55
	2P-F1/B2-10a		0.10			1.00		1.00	0.45
	2P-F1/B2-10b		0.10			1.00		1.00	0.55
	2P-F1/B2-13a		0.13			1.00		1.00	0.45
	2P-F1/B2-13b		0.13			1.00		1.00	0.50
	2P-F1/B2-13c		0.13			1.00		1.00	0.52
	2P-F1/B2-15a		0.15			1.00		1.00	0.40
	2P-F1/B2-15b		0.15			1.00		1.00	0.45
	2P-F1/B2-15c		0.15			1.00		1.00	0.47
	2P-F1/B2-15d		0.15			1.00		1.00	0.50
	2P-F1/B2-20		0.20			1.00		1.00	0.40
1-Part	1P-F9/B14-30b		0.30			1.00		1.00	0.71
	1P-F9/B14-30b			0.09		1.00		1.00	0.90
	1P-F9/B14-35		0.35			1.00		1.00	0.71
	1P-F9/B14-35			0.10		1.00		1.00	0.91
	1P-F1/B2-10b		0.10			1.00		1.00	0.50
	1P-F1/B2-10b			0.03		1.00		1.00	0.57
	1P-F1/B2-20		0.20			1.00		1.00	0.70
	1P-F1/B2-20			0.06		1.00		1.00	0.91
Ratio	1P-F9/B14-30b			0.09		1.00		1.00	0.82
	1PA-F9/B14-30b			0.18		1.00		1.00	0.82
	1PA-F1/B2-10b			0.03		1.00		1.00	0.57
	1PA-F1/B2-10b			0.06		1.00		3.00	1.14

* Total water to meet flowability requirements or water added until segregation occurred.

**Table 4:** Geopolymer CLSM cost analysis.

Mix Design	Cost, min (USD/cy)	Cost, max (USD/cy)	Cost, ave (USD/cy)
F9/B14-20a	10	29	20
F9/B14-30b	14	42	28
F9/B14-35	16	49	33
F9/B14-40b	19	57	38
F9/B14-40c	18	55	37
F9/B14-40d	17	52	35
F1/B2-10b	6	17	11
F1/B2-13c	7	21	14
F1/B2-15b	9	26	17
F1/B2-15d	8	25	16

## Data Availability

The original contributions presented in the study are included in the article, further inquiries can be directed to the corresponding author.
